# Correction: Synthetic routes for a variety of halogenated (chiral) acetic acids from diethyl malonate

**DOI:** 10.1039/c8ra90003e

**Published:** 2018-01-16

**Authors:** Manuel R. Mazenauer, Stole Manov, Vanessa M. Galati, Philipp Kappeler, Jürgen Stohner

**Affiliations:** Institute of Chemistry and Biotechnology, Zurich University of Applied Sciences Einsiedlerstrasse 31 CH-8820 Wädenswil Switzerland sthj@zhaw.ch; Guest Scientist, ETH Zürich, Laboratory for Physical Chemistry CH-8093 Zürich Switzerland just@phys.chem.ethz.ch

## Abstract

Correction for ‘Synthetic routes for a variety of halogenated (chiral) acetic acids from diethyl malonate’ by Manuel R. Mazenauer *et al.*, *RSC Adv.*, 2017, **7**, 55434–55440.

An incorrect email address was provided for affiliation a, the correct version, along with capitalisation of Zürich in affiliation b, is shown below.

The Royal Society of Chemistry apologises for these errors and any consequent inconvenience to authors and readers.

## Supplementary Material

